# Selective Anticancer Properties, Proapoptotic and Antibacterial Potential of Three *Asplenium* Species

**DOI:** 10.3390/plants10061053

**Published:** 2021-05-25

**Authors:** Venelin Petkov, Tsvetelina Batsalova, Plamen Stoyanov, Tsvetelina Mladenova, Desislava Kolchakova, Mariana Argirova, Tsvetanka Raycheva, Balik Dzhambazov

**Affiliations:** 1 Faculty of Biology, University of Plovdiv “Paisii Hilendarski”, 24 Tsar Assen Str., 4000 Plovdiv, Bulgaria; venelin_h_petkov@abv.bg (V.P.); tsvetelina@uni-plovdiv.bg (T.B.); pstoyanov@uni-plovdiv.bg (P.S.); cmladenova@uni-plovdiv.bg (T.M.); kolchakova@uni-plovdiv.bg (D.K.); 2 Faculty of Pharmacy, Medical University of Plovdiv, 15-A Vasil Aprilov Blvd., 4002 Plovdiv, Bulgaria; mariyana.argirova@mu-plovdiv.bg; 3 Faculty of Agronomy, Agricultural University of Plovdiv, 12 Mendeleev Blvd., 4000 Plovdiv, Bulgaria; raicheva@au-plovdiv.bg

**Keywords:** *Asplenium*, anticancer properties, apoptosis, cytotoxicity, superoxide dismutase, antibacterial activity

## Abstract

The ferns *Asplenium ceterach* L., *Asplenium scolopendrium* L. and *Asplenium trichomanes* L. have wide application in traditional medicine worldwide. However, the scientific research on their anticancer and antibacterial properties is insufficient. The present article aims to provide more information on this topic. Extracts derived from the aerial parts of *A. ceterach*, *A. scolopendrium* and *A. trichomanes* were examined using a panel of in vitro assays with different bacterial and mammalian cells. The cytotoxicity and anticancer activity of the samples were analyzed by 3-(4,5-dimethylthiazol-2-yl)-2,5-diphenyltetrazolium bromide (MTT) and Trypan blue assays with three human (A549, FL, HeLa) and three murine (3T3, TIB-71, LS48) cell lines. Inhibitory effects on the growth of Gram-positive (*Bacillus cereus*) and Gram-negative (*Pseudomonas aeruginosa*) bacteria were determined by the agar diffusion assay. Apoptosis-inducing properties of the extracts were analyzed by flow cytometry. Superoxide dismutase (SOD) activity in extract-treated cells was investigated by ELISA. The obtained results demonstrate selective anticancer activity of all three *Asplenium* species. The extract from *A. ceterach* displayed the strongest inhibitory properties against human cervical cancer cells and bacterial cells. It induced a lower level of cytotoxicity against mouse cell lines, indicating a species-specific effect. The extract from *A. trichomanes* demonstrated better anticancer and antibacterial properties than the sample from *A. scolopendrium*. Further experiments linked the mechanism of action of *A. ceterach* extract with oxidative stress-inducing potential and strong proapoptotic potential against the cervical cancer cell line HeLa. *A. trichomanes* and *A. scolopendrium* extracts appeared to be potent inducers of necrotic cell death.

## 1. Introduction

Approximately seven hundred *Asplenium* ferns (family Aspleniaceae, division Pteridophyta) have been identified [1]. The scientific name of this genus and its common name (“spleenwort”) stem from an old doctrine that *Asplenium* plants are useful for treatment of spleen diseases due to their spleen-shaped spores. Indeed, many of these ferns have been used for centuries in traditional medicine in different countries worldwide but their application significantly exceeds therapy of spleen disorders [1,2]. It has been documented that nine *Asplenium* species grow in Bulgaria [3]. Among them are *A. ceterach*, *A. trichomanes* and *A. scolopendrium*—well characterized and widely distributed ferns in Europe. Numerous applications in folk medicine have been reported for these three species. *A. ceterach* has been used for relief of spleen and kidney stones complaints, hemorrhoids, hypertension, respiratory and intestinal disorders, as well as a diuretic, expectorant, laxative, etc. [1,4,5]. *A. scolopendrium* has been used for the treatment of wounds, bleeding, myalgia, lung, liver and spleen diseases, inflammation of gums, urogenital system disorders, and as a mild laxative, diuretic, astringent, diaphoretic and tonic [6,7]. *A. trichomanes* has been applied to relieve cough and renal conditions, as a laxative, expectorant, abortifacient and emmenagogue [8,9]. Its estrogenic activity in vitro has been proven [8], explaining its administration as an emmenagogue.

Plant-derived extracts often represent a rich source of natural compounds with beneficial medical effects. The importance of such substances has been proven since ancient times [10]. Nowadays, purified compounds of plant origins offer a promising alternative to synthetic drugs and have made a major contribution to the therapy of cancer and infectious diseases, but also to the therapy of cardiovascular and autoimmune diseases [10,11]. Their application in drug discovery and development was initiated in the 19th century [10]. Since then, this vast research area continues to expand and present new perspectives for improved pharmaceutical products and development of new drugs with potential to combat incurable diseases [12]. Hence, the present study aims to contribute to this field by investigating the biological activity of three *Asplenium* species growing in Bulgaria—*A. ceterach*, *A. trichomanes* and *A. scolopendrium.* Research on their cytotoxicity against different cell types, as well as anticancer and antibacterial potential, is insufficient. Therefore, the present work aims to unravel this scientific question. Aerial parts from *A. ceterach*, *A. trichomanes* and *A. scolopendrium* growing in Bulgaria were collected and used for preparation of extracts. The obtained samples were analyzed by different in vitro assays with mammalian and bacterial cells. The extracts demonstrated a selective inhibitory effect against a cervical cancer cell line and low cytotoxicity against noncancerous human and mouse cells. *A. ceterach* and *A. trichomanes* extracts displayed antibacterial activity.

The present study extends and complements previous research on *A. ceterach*, *A. trichomanes* and *A. scolopendrium*, which revealed the individual fatty acid composition of the three fern species, as well as the principal bioactive constituents, phenolic acids and flavonoids content of the three *Asplenium* extracts [13]. Our data signify the medicinal interest to the studied three *Asplenium* species, highlight and compare their biological activity.

## 2. Results

### 2.1. Asplenium Ceterach, Asplenium Scolopendrium and Asplenium Trichomanes Extracts Differ in Anticancer Activity and Cytotoxicity against Human and Mouse Cells In Vitro

**Figure 1:**
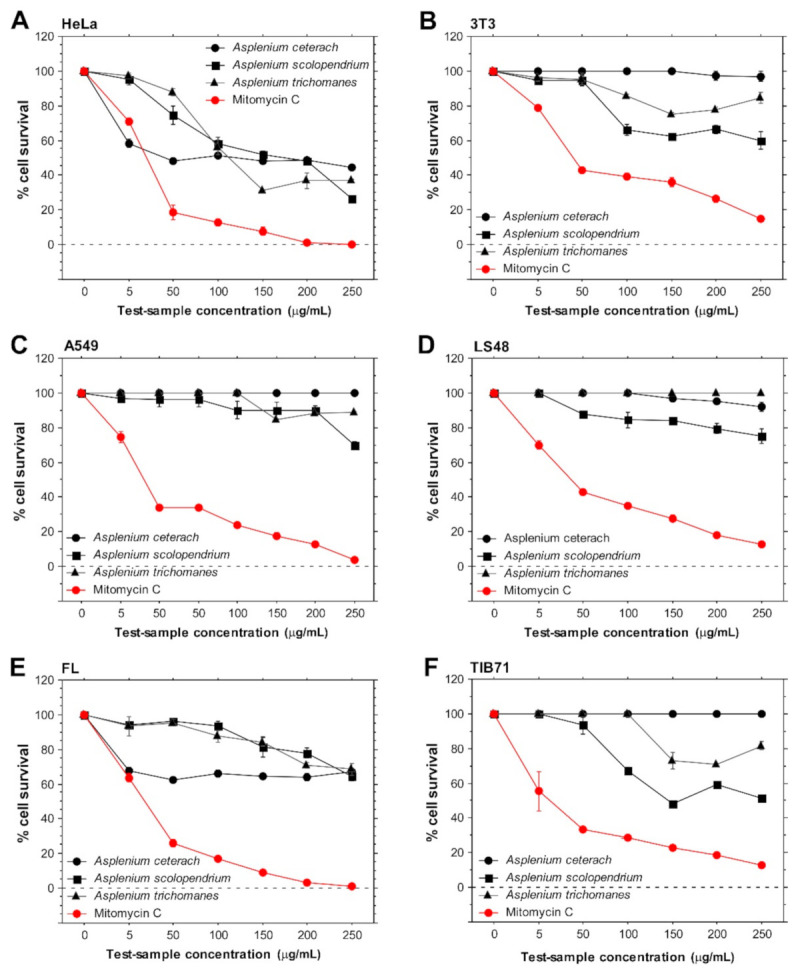
In vitro cytotoxicity and anticancer activity of extracts derived from *A. ceterach*, *A. trichomanes* and *A. scolopendrium*. Cells from six mammalian cell lines were seeded and expanded on 96-well microplates. Then, *Asplenium* extracts of different concentrations were added to the cultures and incubated with the cells for 24 h. The resulting inhibitory effects were measured by MTT assay. Cells treated for 24 h with mitomycin C served as positive control for the assay. The results are expressed as mean percent cell survival (± standard error of the mean). The graphs represent data from two independent experiments. For each experiment, all samples were assayed in triplicates. (**A**,**C**,**E**) show data for human cell lines—HeLa, A549 and FL, respectively. (**B**,**D**,**F**) display results obtained with mouse cell lines—NIH/3T3, LS48 and TIB-71 cells.

The extracts from *A. ceterach*, *A. scolopendrium* and *A. trichomanes* were tested with six different mammalian cell lines in order to determine their in vitro cytotoxicity and anticancer potential. Three human (HeLa, A549, FL) and three mouse (LS48, NIH/3T3, RAW 264.7/denoted as TIB-71/) cell lines were used in the experiments with the aim to investigate whether the extracts induce a general cytotoxic effect against mammalian cells or species-specific cytotoxicity. From the human cell lines, two were cancerous (A549 and HeLa), established from different organs (lung and cervix, respectively), and one was a mouse tumor-derived cell line (TIB-71). The murine LS48 cells were included in the assays due to their high proliferative and invasive activity, similar to cancer cells. The use of this cell line could indicate specific cytotoxic effect against actively dividing cells derived from inflamed tissue that are able to mediate cartilage destruction [14]. Two other noncancerous cell lines (FL, NIH/3T3) served as controls for evaluation of anticancer activity, specifically for human and mouse cells. Based on the chosen panel of cell lines, we were able to evaluate cytotoxic potential against different cell types, species-specific cytotoxicity and inhibitory effects against cancer cells. The obtained results are shown in Figure 1. They demonstrate significant inhibitory effect of all three extracts against HeLa cells. These effects are comparable to the results from mitomycin C treatment—the positive control for the assay with proven antineoplastic properties and suppressive effects on actively proliferating cells. These data indicate that the tested samples possess anticancer activity as they did not exert a strong effect on the growth and survival of noncancerous cell lines (FL, NIH/3T3) (Figure 1B,E). Moreover, the detected anticancer activity was specific for the cervical carcinoma cell line since the development and viability of A549 cells (lung carcinoma) was not considerably affected by incubation with *Asplenium* extracts of different concentrations (Figure 1C).

In addition, *A. ceterach* extract did not affect the growth of all three murine cell lines (Figure 1B,D,F), indicating a species-specific mode of action. Incubation with different concentrations (50–250 μg/mL) of *A. ceterach* extract induced similar levels of inhibition. This observation concerns the only two cell lines affected by treatment with rusty-back fern extract—HeLa and FL, with the HeLa cell line showing the strongest sensitivity. These data suggest that even low concentrations of the extract contain bioactive compounds with significant activity.

The extract from *A. ceterach* displayed the strongest cytotoxic and anticancer potential against human cell lines. Approximately 40 μg/mL of the extract induced 50% inhibition of HeLa cell cultures (Table 1). The samples derived from *A. scolopendrium* and *A. trichomanes* showed significantly higher IC_50_ values.

*A. scolopendrium* extract showed the highest level of cytotoxicity against the three mouse cell lines. The tumor-derived TIB-71 cell line was the most sensitive, with the lowest detected percentage for cell survival. These data indicate species-specific cytotoxicity of *A. scolopendrium*. It also demonstrated antitumor activity as treatment with *A. scolopendrium* extract showed highest inhibition against the tumor-derived mouse cell line. Treatment with *A. trichomanes* extract induced lower reduction in cell survival while incubation with *A. ceterach* did not affect murine cells. The NIH/3T3 cell line was the most sensitive to *A. scolopendrium* and *A. trichomanes* extracts. The intensively proliferating LS48 cells demonstrated the lowest sensitivity to the fern extracts.

**Figure 2:**
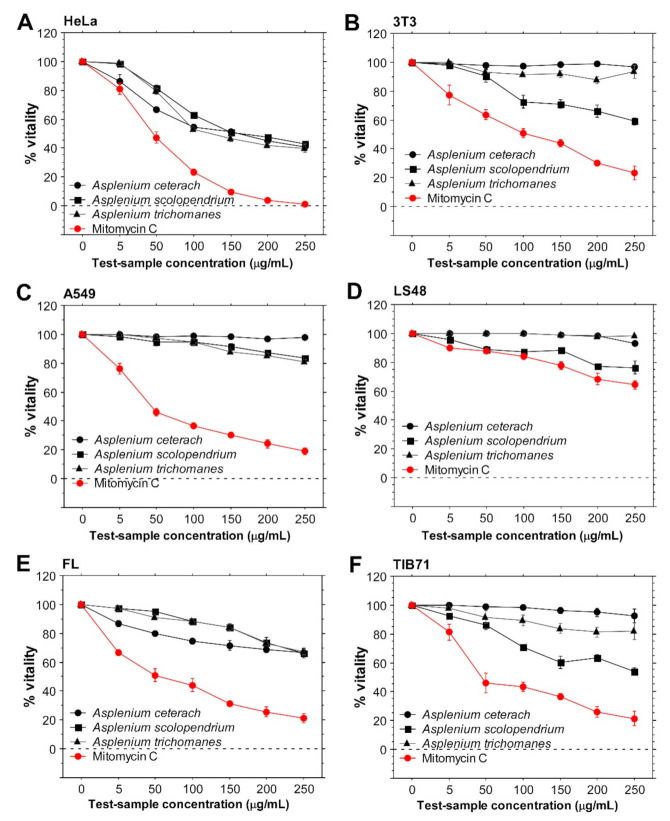
In vitro cellular viability following treatment with extracts derived from *A. ceterach*, *A. trichomanes* and *A. scolopendrium*. Three human and three mouse cell lines were seeded and expanded on 96-well microplates. Then, *Asplenium* extracts of different concentrations were added to the cultures and incubated with the cells for 24 h. The resulting inhibitory effects on cell viability were measured by the Trypan blue assay. Cells treated for 24 h with mitomycin C served as positive control for the assay. The results are expressed as mean percent viability (±standard error of the mean). All samples were assayed in triplicates. (**A**)—results for HeLa cell line, (**B**)—vitality of NIH/3T3 cells following incubation with fern extracts, (**C**)—A549 cells’ viability, (**D**)—results obtained with LS48 cells, (**E**)—data for FL cells. Data shown are for human cell lines—HeLa, A549 and FL, respectively. (**F**) displays results obtained with TIB-71 cells.

The anticancer potential of the examined extracts detected by the MTT method was supported by the Trypan blue vitality assay (Figure 2). The same tendencies were detected, confirming the selective anticancer potential of the three *Asplenium* extracts tested on human cell lines. All extracts reduced the number of viable HeLa cells and again, the *A. ceterach* extract induced the strongest effect. On the other hand, *A. scolopendrium* extract treatment led to marked decrease in viable cell numbers of mouse cell lines, confirming its murine-specific cytotoxicity and higher toxicity against tumor-derived mouse cell line. We used two independent in vitro assays that measure different specific cellular characteristics (viability in Trypan blue assay, cellular reductase activity for MTT assay, respectively) that showed similar results. Therefore, we conclude that the *Asplenium* extracts display selective anticancer activity. They inhibited the viability and metabolic activity of the cervical cancer HeLa cell line. The *A. ceterach* extract showed the strongest effect. The lung adenocarcinoma cell line A549 was not considerably affected by *Asplenium* extract treatment (Figure 2C). FL cells showed inhibition of culture metabolic activity and vitality but it was remarkably lower than the cervical cancer cell line response (Figure 1E, Figure 2E).

### 2.2. Antibacterial Properties of Asplenium Extracts

**Figure 3:**
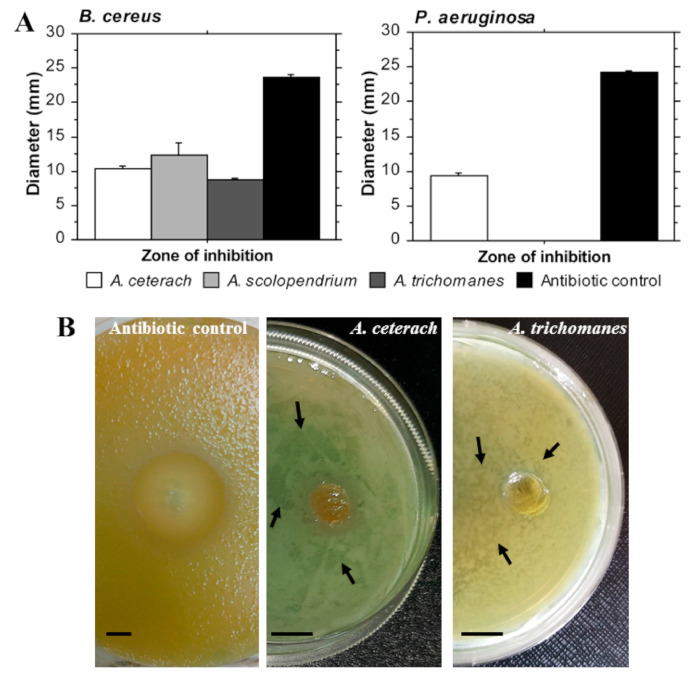
Antibacterial activity of *Asplenium* extracts. Gram-positive (*Bacillus cereus*) and Gram-negative (*Pseudomonas aeruginosa*) bacterial strains were cultured with extract test samples and antibiotic control for 24 h. (**A**)—the figures represent mean diameter (mm) of the zones of bacterial growth inhibition. (**B**)—pictures of *Bacillus cereus* cultures treated with *Asplenium* extracts and antibiotic control. The arrows indicate zones with reduced bacterial growth. The line marks 10 mm length.

One Gram-positive bacterial species (*Bacillus cereus*) and one Gram-negative type (*Pseudomonas aeruginosa*) were chosen for evaluation of potential antibacterial properties of *Asplenium* extracts. The experiments were based on the agar diffusion method and indicated that all three samples inhibit the growth of *B. cereus*, but only *A. ceterach* extract could affect the growth of one Gram-negative species—*P. aeruginosa* (Figure 3A). The measured zones of inhibition were smaller than the antibiotic control, which suppressed the growth of both bacterial strains in 23–25 mm diameter range. Nonetheless, *A. ceterach* and *A. trichomanes* extracts showed prominent effects against *B. cereus* as treatment with these samples led to evident reduction in bacterial growth, which covered a zone with average length of 20–30 mm (Figure 3B). Although it contained limited number of colonies this zone was similar in size with the antibiotic control. Therefore, it could be suggested that bioactive substances from the extracts diffuse in a vast area of the growth medium, hence limiting the bacterial culture development and reducing the number of colonies. Further experiments need to be performed in order to analyze this effect properly, as well as to determine efficiency against antibiotic-resistant strains.

### 2.3. Cell Death-Inducing Properties of Asplenium Extracts

**Figure 4:**
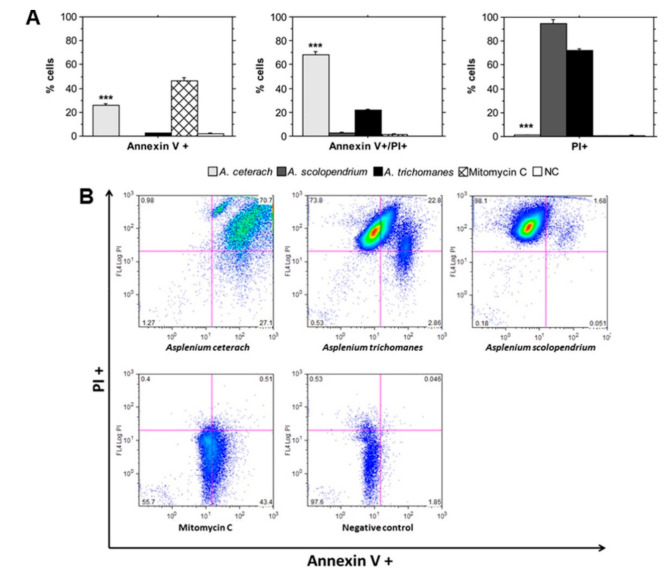
Flow cytometry analysis of apoptotic and necrotic cell populations following treatment with *Asplenium* extracts. HeLa cells were treated with *A. ceterach*, *A. trichmanes*, *A. scolopendrium* extracts and mitomycin-C for 24 h. Control cells were cultured in standard growth medium for the same period. (**A**)—percent annexin V positive, propidium iodide positive, annexin V and propidium iodide double positive cells. Data represent ±SEM. *** *p* ˂ 0.001 was determined by ANOVA. The asterisk marks indicate statistically significant difference between the data for A. ceterach extract and *A. trichomanes* extract, as well as statistically significant difference between the data for A. ceterach extract and A. scolopendrium extract. (**B**)—dot plots representing flow cytometric analyses of HeLa cells stained with annexin V-FITC and propidium iodide (PI).

HeLa cells incubated for 24 h with extracts or mitomycin C were stained with propidium iodide (PI) and Annexin V-FITC and were analyzed by flow cytometry. Figure 4 displays the results from these experiments, indicating significant proapoptotic effect of the extract from *A. ceterach*. Treatment with this sample induced a markedly high percentage of late apoptotic/necroptotic cells. In addition, an elevated number of early apoptotic cells were detected, which was similar to the positive control that contained cells treated with mitomycin C. Accordingly, it may be suggested that the toxic effect of *A. ceterach* extract is mediated by its ability to induce programmed cell death.

Increased percentage of Annexin V/PI double positive cells was measured in the cell sample incubated with extract from *A. trichomanes*. In line with the results for *A. ceterach*, the data on *A. trichomanes* treatment corresponded to the detected cytotoxicity and anticancer activity. The proapoptotic potential of this sample was much lower than one measured for *A. ceterach* in accordance with the difference in the anticancer activity levels and the calculated IC50 values, i.e., higher concentration of *A. trichomanes* is needed in order to achieve 50% inhibition of HeLa cell cultures. 

The extract derived from *A. scolopendrium* did not induce programmed cell death following 24 h incubation with HeLa cells (Figure 4A,B). This result suggests a different mechanism of cytotoxic activity compared to *A. ceterach* extracts. Our hypothesis is supported by the flow cytometry data, which show a high percentage of necrotic (PI positive) cells in the samples treated with *A. scolopendrium* extract. Elevated numbers of PI^+^ cells were also detected in the sample incubated with *A. trichomanes* extract.

### 2.4. A. Ceterach and A. Trichomanes Influence the Activity of SOD in Cervical Cancer Cells

**Figure 5:**
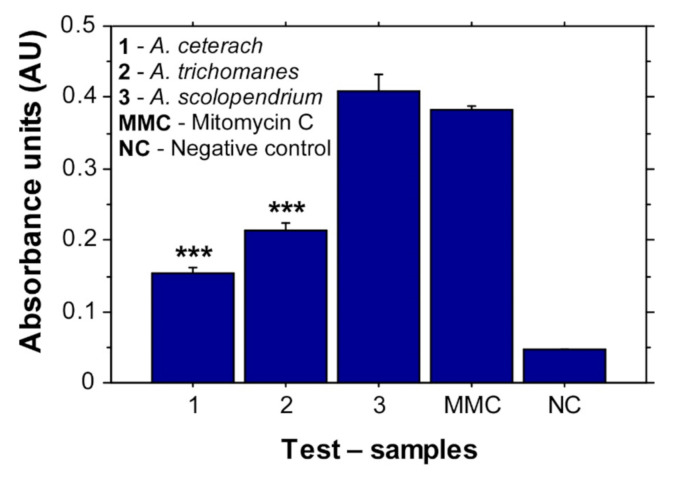
Superoxide dismutase activity levels. Data are presented as mean absorbance units (±SE) measured following 24 h treatment of HeLa cells with *Asplenium* extracts. The detection was based on inhibitory (competitive) ELISA and, thus, reduction in absorbance units levels indicates increased SOD activity. *** *p* ˂ 0.001 was determined by ANOVA. The asterisk marks indicate statistically significant difference between the data for *A. ceterach* extract and Mitomycin C control, as well as statistically significant difference between the data for *A. trichomanes* and Mitomycin C control.

HeLa cells treated with *A. ceterach* and *A. trichomanes* extracts showed increased activity of superoxide dismutase (SOD), which has been considered as a marker for oxidative stress (Figure 5). Again, in accordance with the previous results, the extract from *A. ceterach* induced the most prominent effect. The cells treated with extract from *A. scolopendrium* did not show increased SOD activity. Based on these data, it could be suggested that the extracts from *A. ceterach* and *A. trichomanes* affect HeLa cells by a specific mechanism inducing oxidative stress, which eventually could lead to cell death. These results suggest that the three extracts differ in their mechanisms of action.

## 3. Discussion

The broad applications of *A. ceterach*, *A. trichomanes* and *A. scolopendrium* in traditional medicine have been well described [1,2,4,6,9]. However, the research on their cytotoxicity and inhibitory potential against cancer and/or bacterial cells is insufficient. The data present in this report demonstrate selective anticancer activity for three fern species, showing for the first time that treatment with *A. ceterach*, *A. trichomanes* or *A. scolopendrium* extract inhibits the growth and development of cervical cancer cells in vitro. The results emphasize the highest biological activity of *A. ceterach* extracts among the three studied samples and suggest its utilization as a source of biologically active compounds with selective anticancer and antibacterial activities. Extracts derived from *A. trichomanes* displayed similar properties, but at a significantly lower level. *A. scolopendrium* extracts showed relatively weak anticervical cancer cells activity and selective cytotoxicity in vitro against mouse cell lines and antibacterial properties against Gram-positive bacteria.

The development of multidrug-resistant bacterial strains drives an active search for novel therapeutic compounds with antibacterial activities [15]. Plants are one of the major sources of new substances with bactericidal properties [12]. In the present study, three Asplenium extracts were evaluated for their potential antibacterial activities using two bacterial strains. In accordance with the results for the anticancer activity, the extract from A. ceterach showed the highest antibacterial properties. It inhibited the growth of B. cereus and also the growth of the Gram-negative species—*P. aeruginosa*. *A. trichomanes* extract was also able to induce inhibiting effect on B. cereus but did not affect *P. aeruginosa* cultures. *A. ceterach* and *A. trichomanes* extracts displayed an interesting trend—reduction in bacterial growth in a broader diameter compared to the zone of inhibition. Such an effect has not been reported for these species and it is probably determined by specific low molecular substances in the tested extracts that have higher ability to diffuse in the agar medium. *A. scolopendrium* extract showed inhibitory effect on *B. cereus* growth. These data complement previous findings [13] showing antibacterial potential of the three *Asplenium* extracts against other bacterial strains.

Anticancer properties have been reported for several members of the genus *Asplenium*—*A. nidus*, *A. bulbiferum*, *A. polyodon*, *A. capillus-veneris*, *A. adiantum-nigrum* [15,16,17]. Recently, Fatima et al. (2020) demonstrated that *A. ceterach* exhibits inhibitory activity against prostate cancer cell lines [18]. However, there are no reports about inhibitory properties of this fern against other types of cancer cells. The present article shows for the first time selective inhibitory effects of A. ceterach, A. trichomanes and A. scolopendrium extracts on cervical cancer cells. Comparison of the IC50 values calculated for the three extracts defines A. ceterach extract as the sample with the strongest activity. Thus, our findings support the results for anticancer properties of A. ceterach reported by Fatima et al. (2020) [18,19] and supplement the existing knowledge in the field demonstrating selective inhibitory effect on cervical cancer but no cytotoxicity against the lung cancer cell line A549. In addition, our data are in accordance with the findings of Naghibi et al. (2014) who published pioneering research on the in vitro cytotoxicity of methanolic extracts from A. trichomanes and showed no significant inhibition of lung (A549), hepatic (HepG-2), breast (MCF-7) and colon (HT-29) cancer cells [9]. Hence, our results confirm the lack of inhibition against A549 cells and demonstrate anticancer activity of *A. trichomanes* against cervical cancer cells. The extract from *A. scolopendrium* displayed the weakest anticancer activity in comparison to the other two *Asplenium* extracts but showed the highest cytotoxicity against mouse cell lines. This signifies that species-specific inhibitory properties are probably dependent on specific compounds in this extract. Additional phytochemical studies are needed to support this hypothesis.

The present study contributes not only to the knowledge about the in vitro cytotoxicity of three members of the genus *Asplenium*, but also presents information about the mechanism of their anticancer action. Our results demonstrate that treatment of HeLa cells with *A. ceterach* extract is associated with a significant proapoptotic effect: approximately 30% of the analyzed cellular population displayed an early apoptosis stage and the late apoptosis fraction was double—about 60%. Interestingly, *A. scolopendrium* extract treatment led to a tremendous necrotic cell population increase, indicating a different mechanism of action. In addition, the cell sample incubated with *A. trichomanes* extract showed a high percentage of necrotic cells, but it was lower compared to the *A. scolopendrium*-treated sample. It also showed elevated level of late apoptotic/necroptotic cells. It could be suggested that these effects depend on the biologically active compounds present in the studied samples. Phytochemical analyses of extracts from *A. ceterach* and *A. trichomanes* performed by different research groups have shown significant total phenolic contents and specific composition, determining good antioxidant and other biological activities [4,5,19]. Importantly, both species were shown to produce kaempferol derivatives (*A. ceterach*: kaempferol 3-(6-malonyl)-d-glucoside, kaempferol 3-(6-malonyl)-d-galactoside; *A. trichomanes*: kaempferol 3,7-di-*O*-a-l-rhamnoside, kaempferol 3-*O*-rhamnoside-7-*O*-arabinoside), natural flavonols, which are highly regarded as anticancer molecules [5,20]. The proapoptotic effect of the extracts from *A. ceterach* and *A. trichomanes* could be attributed to the presence of this type of flavonoids as it has been shown that their mechanism of action includes induction of apoptosis [20]. Indeed, the phytochemical analyses of the extracts studied in the present report confirmed the presence of kaempferol in the extracts from *A. ceterach* and *A. trichomanes* [13]. Kaempferol contents in the extract from *A. ceterach* were approximately two times higher than the extract from *A. trichomanes*, which correlates with the detected levels of their anticancer activity. It should be noted that other compounds also contribute to the inhibitory effects of the three *Asplenium* extracts. The individual fatty acid contents of the three fern species were predominated by palmitic acid, ranging from 54 to 66% of all fatty acids analyzed in dried plant material [13]. It has been demonstrated that palmitic acids derived from natural sources have selective antitumor properties [21,22]. Thus, it could be suggested that the significant levels of this fatty acid in the three *Asplenium* species contributes to the selective anticancer effects determined in the present study. Similar roles have been reported for oleic acid [23], which was detected in significant amounts in the studied samples from *A. ceterach*, *A. trichomanes* and *A. scolopendrium* growing in Bulgaria [13]. Comparison of the results from the phytochemical analyses and the present data show a positive correlation between specific fatty acid composition, phenolic acids and flavonoid content of the extracts and their biological activity. In addition, the antioxidant activity of the extracts also correlated with their anticancer and antibacterial properties, with the *A. ceterach* extract showing the strongest potential. The extract from *A. scolopendrium* displayed the lowest activity among the three studied fern species.

Previous phytochemical studies on *A. ceterach*, *A. trichomanes* and *A. scolopendrium* used for preparation of the extracts for the present investigation have shown that *A. ceterach* contains the highest levels of tannins and ω-3 polyunsaturated fatty acids (PUFAs) [13]. Both types of compounds are known for their various positive bioactivities, including anticancer effects [22,24,25]. This fact also supports the highest inhibitory effects detected for *A. ceterach*. Recently, it has been reported that ω-3 PUFA could potentiate the anticancer activity of therapeutic agents by induction of reactive oxygen species (ROS) followed by apoptosis [25]. These findings support our data, which suggest increased ROS levels following treatment with *A. cetera* extracts based on the detected elevated SOD activity. Likewise, ω-3 PUFA in the extracts could synergize and potentiate the inhibitory effects of other specific bioactive compounds. Further examinations are needed to support this hypothesis.

## 4. Materials and Methods

### 4.1. Plant Material

Aerial parts of ferns belonging to the species *Asplenium ceterach* L. (syn. *Ceterach officinarum* Willd*, Ceterach officinarum* DC.; rusty-back fern), *Asplenium scolopendrium* L. (syn. *Phyllitis scolopendrium* (L.) Newman; hart’s tongue fern) and *Asplenium trichomanes* L. (maidenhair spleenwort) were collected during the autumn season from the mountain regions in South and Central Bulgaria [13]. The material from *A. ceterach* was collected from the region of Asen’s Fortress, Rhodope Mountain, Southern Bulgaria. Aerial parts from *A. scolopendrium* and *A. trichomanes* were collected in the National park “Bulgarka”, Balkan mountain range, Central Bulgaria. The species were identified by Assoc. Prof. Plamen Stoyanov (Department of Botany, Faculty of Biology, University of Plovdiv “Paisii Hilendarski”) and voucher specimens No. 062643, No. 062644 and No. 062649, respectively, were deposited in the Herbarium of the Agricultural University of Plovdiv (SOA). The plant material was air dried at ambient temperature (23–25 °C) in darkness in order to prevent degradation of light-sensitive compounds. Prior to the extraction procedure, the dried material was powdered using glass mortar.

### 4.2. Preparation of Plant Extracts

Extracts from each *Asplenium* fern were prepared using 10 g powdered plant material. The procedure included maceration of plant powder in 250 mL 70% aqueous solution of methanol for 24 h on a magnetic stirrer in dark place at room temperature. After centrifugation (6000 g for 10 min), the resulting extracts were filtered through Whatman No. 1 filter paper (Sigma-Aldrich, Steinheim, Germany). The procedure was repeated two more times under the same conditions. Then, the extracts from each species were pooled and methanol was removed by evaporation under vacuum at 37 °C using Savant (SAVANT Instruments Inc., Farmingdale, NY, USA). The dried extracts were dissolved in 50% DMSO aqueous solution (*w*/*v*) to a final concentration of 5 mg/mL. All extracts were stored at 4 °C in darkness until further analysis.

### 4.3. Cell Lines and Culture Conditions

Three human and three murine cell lines with anchorage-dependent growth were used in the present study. These are: A549 (ATCC^®^ CCL-185™), human lung carcinoma origin; HeLa (ATCC^®^ CCL-2™), human cervical adenocarcinoma cell line; FL (NBIMCC 94), human amnion origin; RAW 264.7 (ATCC^®^ TIB-71™), denoted as TIB-71, mouse monocyte/macrophage cell line derived from Abelson murine leukemia virus-induced tumor; NIH/3T3 (ATCC^®^ CRL-1658™), mouse embryonic fibroblasts; LS48 (DSM ACC 2455; Biotectid, Germany) mouse fibroblast-like permanent cell line.

Cultures from all cell lines were grown in Dulbecco’s modified Eagle’s medium (DMEM) (Sigma-Aldrich Inc., Merck KGaA, Darmstadt, Germany) supplemented with 10% heat-inactivated fetal bovine serum (FBS) (Sigma-Aldrich Inc., Merck KGaA, Darmstadt, Germany), 100 U/mL penicillin and 100 μg/mL streptomycin (Sigma-Aldrich Inc., Merck KGaA, Darmstadt, Germany). This growth medium is denoted as complete DMEM. The cells were cultured under sterile conditions in a humidified incubator, maintaining a 37 °C temperature and 95% atmospheric air/5% CO_2_ content. The cultures were expanded in 75 cm^2^ flasks (TPP, Trasadingen, Switzerland). At 80–90% confluency, the cells were trypsinized and cell vitality was determined by the Trypan blue assay [26].

### 4.4. In Vitro Cytotoxicity and Anticancer Activity Assays

Following trypsinization and live cell count assessment, suspensions with concentration 1 × 10^5^ cells/mL were prepared. Then, 200 μL cell suspension/well was plated on 96-well plates (TPP, Trasadingen, Switzerland) and cultured for 24 h under standard conditions (37 °C, 5% CO_2_ content and high humidity). Then, the cells were incubated with *Asplenium* extracts for 24 h. Different extract concentrations were assayed—5, 50, 100, 150, 200 and 250 µg/mL. The extracts were diluted in complete DMEM. Untreated cells cultured for 24 h in complete DMEM served as negative control. Cells incubated for 24 h with mitomycin C (Sigma-Aldrich Inc., Merck KGaA, Darmstadt, Germany) were used as a positive control. Another control included cells treated for 24 h with different concentrations of DMSO (2.5%, 2%, 1.5%, 1%, 0.5%, 0.05%). These concentrations corresponded to the level of DMSO in the tested samples (250, 200, 150, 100, 50, 5 µg/mL extract, respectively). All samples were assayed in triplicates. Two independent experiments were performed. 

Cytotoxicity and anticancer activity in vitro were determined using the Trypan blue and MTT assay. The MTT assay was based on the method described by Edmondson with slight modifications [27]. In brief, at the end of the 24 h incubation period with fern extracts, the culture medium in all test wells was removed and 100 μL/well 0.5 mg/mL MMT ([3-(4,5-dimethylthiazol-2-yl)-2,5-diphenyltetrazolium bromide]) (Sigma-Aldrich Inc., Merck KGaA, Darmstadt, Germany) solution was added to the cell culture plates and incubated for 2 h at 37 °C in darkness. After that, the MTT solution was removed and 100 μL/well DMSO (Sigma-Aldrich Inc., Merck KGaA, Darmstadt, Germany) were pipetted into each well. The plates were incubated for 10–15 min at 37 °C in order to extract the formazan accumulated in the cells. Then, absorbance at 570 nm was measured using Synergy-2 microplate reader (BioTek, Winooski, VT, USA). Percent cell survival and percent inhibition of cell vitality and development were calculated using the data from extract-treated cells and cells cultured in standard conditions without test sample. Percentages of inhibition induced by the DMSO controls were subtracted from the values of the corresponding samples. IC_50_ values were calculated based on the data for percent inhibition.

Trypan blue staining was performed to determine the number of viable cells following treatment with *Asplenium* extracts. The assay was based on the method described by Strober et al. [26]. In our experiments, after 24 h incubation with test extracts, the cells were detached by trypsinization. Then, 50 μL cell suspension was mixed with equal volume of Trypan blue (Sigma-Aldrich Inc., Merck KGaA, Darmstadt, Germany). The Trypan blue dye cannot penetrate through the intact membranes of viable cells. Thus, it stains only the dead cells in the examined cell suspension. The stained sample was loaded on a Burker chamber (Boeco, Hamburg, Germany) and the viable cells were counted using a microscope (Ceti Max III compound microscope, Medline scientific, Oxon, UK); concentration of live cells per mL of the sample was calculated. Percent vitality was determined based on the concentration of viable cells in the extract-treated cell cultures and the concentration of viable cells in the control samples treated with corresponding concentration of DMSO. All samples were analyzed in triplicates.

### 4.5. Analysis of Antibacterial Activity In Vitro

The antibacterial activity of the three *Asplenium* extracts was determined by the agar diffusion method [28]. One Gram-negative (*Pseudomonas aeruginosa* ATCC^®^ 27853™) bacterial strain and one Gram-positive (*Bacillus cereus* ATCC^®^ 11778™) bacterial strain were used in the study. Bacteria were grown from glycerol stocks stored at −80 °C. In total, 25 μL of thawed bacterial stocks were plated on nutrient agar and grown overnight at 37 °C. Following thawing and culture on solid medium, one colony was inoculated in nutrient broth and incubated at 37 °C for 24 h. Mueller Hinton medium (agar or broth) (Sigma-Aldrich Inc., Merck KGaA, Darmstadt, Germany) was used to culture *P. aeruginosa* and *B. cereus*. For determination of antibacterial activity, petri dish plates with nutrient agar were inoculated with 50 μL bacterial suspension with a concentration of 1 × 10^6^ CFU/mL. Then, 20 μL of test samples containing 20 μg *Asplenium* extract was applied on the plates. They were placed in standard holes in the agar medium with diameters of 8 mm. Buffer solution that contained 20 IU penicillin and 20 μg streptomycin served as a positive control. The plates were incubated with test samples for 24 h at 37 °C and then the average diameter of bacterial growth inhibition zones was measured. All samples were analyzed in duplicates.

### 4.6. Determination of SOD Activity

Superoxide dismutase (SOD) activity in HeLa cells treated with 20 μg/mL *Asplenium* extracts or 100 μg/mL mitomycin C for 24 h was determined using a SOD Determination Kit (Sigma-Aldrich Inc., Merck KGaA, Darmstadt, Germany). The assays were performed using the protocol recommended by the manufacturer. SOD activity measurement was based on a colorimetric method that utilizes the highly water-soluble tetrazolium salt, WST-1 [2-(4-iodophenyl)-3-(4-nitrophenyl)-5-(2,4-disulfophenyl)-2H-tetrazolium monosodium salt]. Upon reduction with a superoxide anion, WST-1 produces a water-soluble formazan dye that can be measured spectrophotometrically based on absorbance at 450 nm. The values of the detected absorbance units are proportional to the quantity of superoxide anion. Thus, SOD activity was measured as an inhibitory effect on absorbance of the assayed samples. Absorbance was measured using a Synergy-2 microplate reader (BioTek, Winooski, VT, USA). All samples were assayed in triplicates.

### 4.7. Estimation of Apoptosis 

The potential proapoptotic effect of *Asplenium ceterach*, *Asplenium trichomanes* and *Asplenium scolopendrium* extracts was investigated by staining with Annexin V-FITC and propidium iodide (PI) followed by flow cytometric analysis. In brief, 1 × 10^5^ HeLa cells/mL were seeded on 6-well plates (TPP, Trasadingen, Switzerland) and cultured under standard conditions for 24 h. Then, the growth medium was replaced with equal volume complete DMEM medium containing 250 μg/mL *Asplenium* extract and the cells were cultured for another 24 h. Cells treated with 100 μg/mL mitomycin C served as a positive control for the assay. At the end of the respective treatment, the cells were harvested, centrifuged and resuspended in 500 μL binding buffer. Afterwards, 5 μL Annexin V-FITC (Abcam, Cambridge, UK) and 5 μL PI (Abcam, Cambridge, UK) were added to each sample and then the cells were incubated for 20 min at room temperature in darkness. Then, the cells were washed and apoptosis rates were analyzed by flow cytometry using a Cytomics FC 500 Flow Cytometer (Beckman Coulter, Brea, CA, USA). All samples were analyzed in duplicates.

### 4.8. Statistics 

ANOVA was applied for statistical analyses using the StatView software (SAS Institute Inc., Cary, NC, USA). The displayed statistically significant differences between samples groups were determined by Fisher’s PLSD. Calculated *p* values lower than 0.05 were considered statistically significant.

## 5. Conclusions

The present study demonstrates selective anticancer potential of three *Asplenium* species, among which *A. ceterach* showed the most potent activity. The reported results bring new information on the mechanism of anticancer activity induced by treatment with extracts from *A. ceterach*, *A. trichomanes* and *A. scolopendrium*. The inhibiting effect against the cervical cancer cell line HeLa following exposition to *A. ceterach* extract was associated with increased SOD activity and a proapoptotic mechanism. Treatment with *A. trichomanes* extract also resulted in increased SOD activity, but the effect was milder compared to the sample from *A. ceterach*. Instead of a proapoptotic effect, A*. trichomanes* and *A. scolopendrium* samples induced necrosis in HeLa cell populations, indicating a different mechanism of anticancer activity. Further research on these extracts, especially the one from *A. ceterach*, could identify new compounds with anticancer and antibacterial activities. Thus, this fern species could represent a source of bioactive substances for production of more effective pharmaceutical products for treatment of cancer and infectious diseases.

## Figures and Tables

**Table 1:** IC_50_ determined following 24 h treatment with *Asplenium* extracts.

Extract	IC_50_ (μg/mL)
*Asplenium ceterach*	40.48 ± 6.47 ***
*Asplenium trichomanes*	120.68 ± 4.7
*Asplenium scolopendrium*	204.83 ± 3.6

The data are shown as mean ± SE and represent extract concentrations (μg/mL) that inhibit 50% of HeLa cell cultures metabolic activity and vitality, measured by MTT assay. *** *p* ˂ 0.001 was determined by ANOVA. The asterisk marks indicate statistically significant difference between the data for *A. ceterach extract* and *A. trichomanes* extract, as well as statistically significant difference between the data for *A. ceterach* extract and *A. scolopendrium* extract.

## Data Availability

Data is contained within the article.
